# Navigating the Factors Affecting Functional Impairment in Spondyloarthritis

**DOI:** 10.18295/squmj.1.2024.005

**Published:** 2024-05-27

**Authors:** Kaouther Maatallah, Ines Cherif, Hanen Ferjani, Dorra Ben Nessib, Rania Boumaiza, Dhia Kaffel, Wafa Hamdi

**Affiliations:** University Tunis El Manar, Faculty of Medicine, Tunis, Tunisia; Kassab Institute of Orthopedics, Manubah, Tunisia

**Keywords:** Spondyloarthritis, North Africa, Tunisia

## Abstract

**Objectives:**

This study aimed to assess the predictive factors of functional impairment in spondyloarthritis (SpA) patients assessed with bath ankylosing spondylitis functional index (BASFI) and Lequesne Index (LI).

**Methods:**

This retrospective study was conducted at the Rheumatology Department of Mohamed Kassab Institute of Orthopedics, Manubah, Tunisia, and collected data from 2008 to 2019 over a period of 4 months (August to November 2019). Socio-demographic and disease-related data of SpA patients were collected. Disease activity was assessed using the bath ankylosing spondylitis-global score (BASG-s) and the bath ankylosing spondylitis disease activity index (BASDAI). The spinal mobility was evaluated by the bath ankylosing spondylitis metrology index (BASMI). Structural progression was evaluated with the bath ankylosing spondylitis radiologic index (BASRI) and modified stoke ankylosing spondylitis spine score (mSASSS). A multivariate analysis was done to search for predictive factors associated with BASFI and LI.

**Results:**

A total of 263 patients were included. The mean age was 38.9 ± 12.7 years and the gender ratio was 2.7. The mean age of onset of SpA was 27.6 ± 10.8 years and disease duration was 11.3 ± 9.5 years. Occupation was significantly associated with BASFI and LI scores. A significant functional impact was notably correlated with a long duration of the disease. The two scores were correlated with a limitation of spinal mobility (BASMI), a greater disease activity (BASDAI and erythrocyte sedimentation rate) and a greater impact of the disease on health status (BASG-s). Significant functional impairment was also correlated with structural impairment (mSASSS, BASRI and sacroiliitis grade). The variables independently related to BASFI were the mSASSS score and the BASDAI. The variables independently related to LI were profession (unemployed subjects had higher scores), the mSASSS score and the BASMI.

**Conclusion:**

Occupation, disease activity, mobility and structural progression predicted functional impairment in Tunisian SpA patients.

**Advances in Knowledge**

- *This study provides a thorough evaluation of functional impairment in spondyloarthritis (SpA) patients, utilising both the bath ankylosing spondylitis functional index and Lequesne Index (LI), shedding light on the underexplored LI in the context of SpA.*
- *Furthermore, it identifies a wide range of factors influencing functional impairment, including disease duration, occupation, disease activity, spinal mobility, structural progression and health status, providing a comprehensive view of the multifaceted nature of SpA.*
- *This research focuses on North African SpA patients, contributing valuable insights into the specific factors affecting this population, which may have distinct characteristics compared to other populations.*

**Application to Patient Care**

- *The identification of disease duration as a significant factor highlights the importance of early diagnosis and intervention to minimise functional impairment and enhance patients’ quality of life.*
- *Healthcare providers can use the insights from this study to adopt a holistic approach to SpA management, considering disease activity as well as mobility, structural progression and patient-reported outcomes.*
- *The study’s focus on North African SpA patients facilitates culturally sensitive and region-specific care, ensuring that interventions align with the unique needs of this population.*

Spondyloarthritis (spa) is a chronic inflammatory rheumatic disease that occurs particularly in young adult males. The prevalence of this disease varies across regions and its data regarding North Africa and the Middle East is limited.^1^ North African patients with SpA exhibit distinct clinical and genetic features. North African patients tend to have more severe forms of SpA, with a higher prevalence of the axial form and coxitis.^1^^,^^2^ Another important feature is the genetic trait of North African SpA patients with very low prevalence of human leukocyte antigen-B27.^3^

The occurrence of SpA in a young and active subject makes this disease an extremely challenging one. The perception of SpA has shifted from a debilitating and disabling disease to one that can be managed and controlled with appropriate advanced therapy. Although remarkable progress has been made in the past decades in the field of pathophysiology, there is still a long way to go; patients are still plaintive and report symptoms of pain fatigue and functional impairment, which are key patient-related outcomes.^4^

Patient age, gender, ethnicity, education level, smoking status, disease duration, medication use and family history of arthritis and inflammation have emerged as potential risk factors for functional limitation in the literature.^5^ Functional impairment is also associated with increased levels of work disability and a decrease in work productivity.^6^ This, in turn, results in considerable economic and societal costs.^7^ Considering these consequences, it is crucial to investigate the factors contributing to functional impairment among SpA patients.

This study aimed to evaluate the functional impact in Tunisian SpA patients and identify the factors that are most correlated with the alteration of their functional prognosis. These insights can be leveraged to develop targeted interventions that can improve the quality of life and function of SpA patients. This will contribute to individual well-being as well as the overall economy and society.

## Methods

This retrospective study included SpA patients who fulfilled the Assessment of Spondyloarthritis International Society (ASAS) criteria and were followed at the Rheumatology Department of the Mohamed Kassab Institute of Orthopedics, Manubah, Tunisia, between 2008 and 2019, with data collection spread over 4 months (August 2019 to November 2019). A data collection form was created and relevant information was extracted from medical records. Patients with an associated chronic inflammatory rheumatism and those with pre-existing disabling conditions (pre-existing disability independent of the disease and its complications) were not included to limit confounding factors.

Socio-demographic data was collected as well as clinical data included the age of onset of the disease, the duration of SpA and the presence of spinal pain and coxitis. The search for extra-articular manifestations was recorded. Disease activity, functional impact and spinal mobility were assessed using specific tools. Disease activity was assessed by inflammatory biomarkers (erythrocyte sedimentation rate [ESR] and C-reactive protein), the bath ankylosing spondylitisglobal score (BASG-s) and the bath ankylosing spondylitis disease activity index (BASDAI). The assessment of functional discomfort in SpA patients was scored using the bath ankylosing spondylitis functional index (BASFI) and the hip Lequesne Index (LI). The presence of pain at enthesis sites was evaluated by the Maastricht ankylosing spondylitis enthesitis score (MASES). Spinal mobility was evaluated by the bath ankylosing spondylitis metrology index (BASMI).

Assessment of structural involvement was determined by the bath ankylosing spondylitis radiologic index (BASRI) and modified stoke ankylosing spondylitis spine score (mSASSS). The grading of sacroiliitis was done using the modified New York criteria.^8^^,^^9^ The radiological form of coxitis was specified (early coxitis, enveloping coxitis, pseudoarthritic coxitis, destructive coxitis and synostotic coxitis) and therapeutic modalities were provided.

All calculations were made using the Statistical Package for Social Sciences (SPSS), Version 25 (IBM Corp., Armonk, New York, USA). For the analytical study, the qualitative variables were compared by the analysis of variance with 1 factor. The study of correlation was performed by Pearson’s correlation coefficient. To find the variables independently linked to the BASFI and the LI, a multivariate analysis was conducted in multiple linear regression, step-by-step descending method (in the first step, all the variables were introduced and from step-to-step, the variable with the least significant *P* was removed). Significance was accepted for a *P* <0.05 for all statistical tests.

All patients provided consent to participate. The study was approved by the ethics committee of Kassab Institute Ethical Committee (IMKO018). This study was carried out per the guidelines in the Declaration of Helsinki and STROBE guidelines were followed.

## Results

A total of 263 patients were included in this study. The mean age of the patients was 38.9 ± 12.7 years (range: 16–79); 192 were men (73.0%) and 71 women (27.0%). The mean age of onset of SpA was 27.6 ± 10.8 years (range: 5–61) and the disease duration was 11.3 ± 9.5 years (range: 1–62). According to ASAS criteria, SpA was axial in 43.0% of cases and peripheral in 43.7% of cases with enthesitic involvement in 19.1% of cases. SpA was axial and peripheral in 44.5% of cases [Tables 1 and 2].

Occupation was significantly associated with BASFI score and LI. The post hoc test showed a higher BASFI (*P* = 0.010) and LI score (*P* < 0.001) in the unemployed subjects. Regarding baseline disease-related features, a significant functional impact was notably correlated with a long duration of the disease for BASFI (*P* = 0.007) and LI (*P* <0.001).

Peripheral involvement was also significantly associated with BASFI and LI score (*P* = 0.006 and *P* = 0.021, respectively). Extra-articular involvement was not associated with higher functional impairment (*P* = 0.907 for BASFI and *P* = 0.152 for LI). Neck and back pain were significantly associated with BASFI and LI scores. The same was true for coxitis, its bilateral character and the limitation of hip mobility [Table 3].

The two scores were correlated with a limitation of spinal mobility (BASMI), a greater disease activity (BASDAI and ESR) and a greater impact of the disease on health status (BASG-s) [Table 4].

Regarding the radiological data, radiological forms of coxitis (*P* = 0.037 for BASFI and *P* <0.001 for LI) were significantly associated with functional discomfort. The post hoc test showed that a destructive form of coxitis was associated with a higher BASFI and LI score.

Significant functional impairment was also correlated with structural impairment: mSASSS (*P* = 0.001 for BASFI and *P* = 0.003 for LI), BASRI (*P* <0.001 for both BASFI and LI) and sacroiliitis grade (*P* <0.001 for both BASFI and LI).

The variables independently related to BASFI were the mSASSS score and the BASDAI.

The variables independently related to LI were profession (unemployed subjects had higher scores), the mSASSS score and the BASMI [Table 5].

## Discussion

This study aimed to determine the main factors associated with significant functional impact in North African SpA patients. BASFI and LI were higher in unemployed patients. Moreover, greater functional impact was associated with peripheral involvement, neck pain, back pain and coxitis. The elevation of both scores was also significantly correlated with a longer duration of disease progression. The BASFI and LI were correlated with a limitation of spinal mobility and greater disease activity. BASFI correlated with a higher MASES score and the presence of low back pain.

Regarding the association between radiological data and scores assessing functional discomfort, a high BASFI and LI were significantly associated with the presence of a destructive form of coxitis. They were also correlated with structural damage (mSASSS, BASRI and sacroiliitis grade).

To the best of the researchers’ knowledge, there have been very few studies in the literature that have evaluated the factors associated with a high LI, a French score developed in the 80s, in SpA patients.^10^ A total of 2 published articles were found during the current study’s literature review that used the LI to assess functional impairment in SpA patients.^11^^,^^12^ Both articles were from Tunisia, a francophone country. This highlights one particularity of this study—the search for all potential factors associated with LI in a population where it has been little used. However, in practice, this index is frequently used to assess the functional impact of hip and knee damage in osteoarthritis patients. A strength of the current study is that it searched for all potential demographical, clinical and para-clinical factors associated with functional impairment in a population of SpA patients known to have a higher prevalence of coxitis. However, the retrospective nature may be a limitation. Furthermore, the disease was active (BASDAI >40) in 65.6% of cases. This could be explained by a recruitment bias because this study was carried out in a university hospital that manages severe forms of SpA.

Regarding the socio-demographic features, the profession of the patient was significantly associated with the LI as well as the BASFI score. It was, indeed, independently associated with LI, with higher values in the unemployed subjects. The absence of professional activity would be the consequence of an important functional impact of the disease and not its cause. On the other hand, in Ward *et al*.’s study each increase in the occupational physical activity score—a score that calculates the average level of activity of each job the patient has held in his or her lifetime (1 mild, 2 moderate, 3 intense)—adjusted by the number of years spent in each job (score between 1 and 3), increased the BASFI by 8.9 points.^5^ ‘Occupation’ emerged as an important health-related outcome in SpA in the current study. The bulk of studies showed that the prevalence of work disability is high in these patients and is associated with both clinical and psychosocial factors.^13^^,^^14^ Specifically, labour-intensive jobs and manual professions were associated with poorer work outcomes.^13^^,^^15^ Ulus *et al*. compared ankylosing spondylitis patients with healthy controls and found that functional impairment assessed by BASFI was a significant predictor of work instability scores.^16^ The current study found that unemployed patients had higher scores on the BASFI and LI measures. This observation may be attributed to their inability to maintain employment due to functional limitations.

In terms of clinical examination, peripheral involvement was also significantly associated with LI and BASFI scores. Indeed, the more enthesitic involvement and the more painful and swollen the joints are, the higher the BASFI score.^17^ Coxitis was particularly associated with a higher LI and BASFI. This is in-line with the literature.^18^^,^^19^ It increased by 1.6 compared to patients without coxitis, and higher values were found for all BASFI questions that seemed to assess the functional impact on the hips (e.g. difficulty in getting up from the floor or a chair, tying shoes and climbing stairs), as well as for those questions that did not assess gestures involving the hips such as looking over the shoulder without turning around.^18^ According to a recent study of patients with axial SpA, it has been suggested that hip involvement has a greater impact on functional disability than axial structural damage. In addition, coxitis may affect the ability to accommodate a rigid lumbar spine, which may exacerbate functional limitations.^20^ Coxitis remains a major and dreaded prognostic location in SpA patients, especially in the North African population where it exists in higher prevalence.^2^ In this study, more than half the patients had coxitis. Fortunately, the treatment response seems similar to that of Western countries.^2^ These data underline the importance of early management of coxitis. As the hip is a weight-bearing joint, its damage affects not only the patients’ daily movements but also walking and thus the entire spinal statics. Hence, it would contribute to the aggravation of the deformities already present.

In this study, disease activity, as assessed by the BASG-s, BASDAI and ESR, was significantly correlated with LI. The same was true for the BASFI score. The association with BASDAI was found after multivariate analysis by linear regression, which underlines the pivotal role of this factor in the functional impact of the disease. Studies have supported the results of this analysis stating that BASDAI was independently associated with BASFI.^21^^,^^22^ The observed increase in functional impairment among patients with higher disease activity may be in part explained by a concomitant decrease in spinal mobility. BASDAI was reported to be associated with BASMI.^23^

The current study found that the BASMI was significantly associated with LI and BASFI scores, which has also been reported by other studies in the literature.^24^ These data show that axial mobility limitation is closely related to function in these patients. More importantly, the BASMI was independently associated with the LI after multivariate linear regression in this study. Spine mobility is a key clinical feature that should be assessed at baseline. It is strongly dependent on both inflammatory activity and structural damage, and the BASMI was proved to be more contributory in assessing this parameter compared to other scores.^23^ Moreover, results from the Carvalho *et al*.’s study show that spinal inflammation is independently and positively associated with BASMI.^25^

Inflammation, assessed with ESR values, has a significant anabolic effect on bone in SpA. Based on longitudinal data, it appears that effectively managing inflammation may decelerate the radiographic advancement of axial SpA.^26^

This appears to be even more relevant as an association of both BASFI and LI with mSASSS was found after multivariate analysis by linear regression in the current study. Structural damage leads to decreased spinal mobility and difficulty in carrying out daily tasks. Studies have concurred with the results of this analysis stating that mSASSS was independently associated with BASFI.^21^ Ankylosis in SpA is the latest stage of structural damage. Although the natural course of the disease has changed positively over the past years, joint ankylosis is not rare and affects 20–50% of patients.^26^ This study assessed the radiographic structural damage using mSASSS. However, this score measures anterior damage of the vertebrae and underestimates postero-lateral vertebral rim ankylosis such as ankylosis of the facet joint. Jung *et al*. showed that facet joint ankylosis may be more associated with functional impairment than syndesmophytes.^26^ Consequently, both anterior and posterior structural damage should be investigated to gain a better understanding of the patient’s condition.^27^

The grade of sacroiliitis was positively correlated with both BASFI and LI in this study. The impact of sacroiliitis on clinical outcomes and functional status has been supported by others.^28^^,^^29^ In the German SpA inception cohort, structural damage of the sacroiliac joints influenced functional status and spinal mobility. The results suggested that an increase of one grade of radiographic sacroiliitis in a single joint is associated with a deterioration of 0.10/0.12 points in BASFI/BASMI, respectively, regardless of disease activity and structural damage in the spine.^28^ As for the therapeutic modalities, neither non-steroidal anti-inflammatory drugs (NSAIDS) nor disease-modifying antirheumatic drugs (DMARDS) appeared in the multiple regression analysis.

Several studies in the literature have shown the involvement of NSAIDs in functional impairment. This was found in a study by Kroon *et al*. where the mean BASFI in the NSAID group decreased by 9.1 points (5.1–13) compared with the no-treatment group.^30^ However, a few studies have evaluated the effect of conventional DMARDs on the functional impact of the disease. In a Turkish case-control study, which included 51 patients with ankylosing spondylitis, no improvement in BASFI score was noted when comparing patients receiving NSAIDs alone to those receiving them in combination with methotrexate.^31^ Similarly, no improvement in BASFI score was found after 16 weeks of methotrexate.^32^ However, Gonzalez-Lopez *et al*. found that the group receiving methotrexate had a better BASFI than the group receiving the placebo.^33^

Tumour necrosis factor (TNF) alpha inhibitors did not influence the functional outcome of SpA in the present study, whereas several researchers have reported the beneficial effect of anti-TNF alpha on function during SpA.^34^ This could be explained by the fact that only 7.76% of patients in the study received TNF alpha inhibitors.

This study has some strengths including the sample size, which provides a good basis for reliable statistical analysis and the use of functional indexes that have been infrequently utilised in similar studies. Furthermore, the disease duration of patients in this series ranged from 1 to 62 years, enabling a credible assessment of functional impact. However, it is important to state the weaknesses of this research. First, the retrospective nature of the study does not allow researchers to establish a causal link and draw solid conclusions. However, there was very little missing data in the files. Finally, the disease was active (BASDAI >40) in 65.6% of cases. This could be explained by a recruitment bias, as the study was carried out in a university hospital treating severe forms of SpA.

## Conclusion

The findings of this study are consistent with the existing epidemiological data, which report a high prevalence of coxitis in North African patients with SpA. Employment status, spinal mobility, disease activity and structural damage emerged as predictive factors independently associated with functional impairment. The LI, more frequently used in European and French-speaking countries, has been used in particular in osteoarthritis patients and has proven its relevance and usefulness in SpA patients. Early and effective treatment is crucial for achieving rapid and sustained remission in SpA. This should be defined, ideally, as remission across all clinical, biological and radiological domains. Emerging therapies are targeting all 3 domains and have, therefore, revolutionised the management of SpA, providing new opportunities to optimise patient outcomes. Prospective studies with a long-term follow-up of the patients are needed for a better evaluation of the effect of these new therapeutic modalities on the functional impact of the disease.

## Figures and Tables

**Table 1:** Socio-demographic and disease-related characteristics of patients with spondyloarthritis (N = 263)

Characteristic	n (%)
**Mean age in years ± SD (range)**	38.9 ± 12.7 (16–76)
**Gender ratio**	2.7
**Status**	
Single	119 (45.2)
Married	136 (51.7)
Divorced	6 (2.3)
Widow(er)	2 (0.8)
**Level of education**	
Illiterate	33 (12.5)
Primary level	95 (36.1)
Secondary level	95 (36.1)
Higher level (university)	40 (15.2)
**Professional occupation**	
Salaried employment	116 (44.1)
Self-employed	27 (10.3)
Unemployed	92 (35.0)
Student	18 (6.8)
Retired	10 (3.8)
**Social class**	
Disadvantaged	62 (23.6)
Middle class	174 (66.2)
Privileged	27 (10.3)
**Mean age at onset in years ± SD (range)**	27.6 ± 10.8 (5–61)
**Mean disease duration in years ± SD (range)**	11.3 ± 9.5 (1–62)
**Classification of SpA**	
Axial	113 (43.0)
Peripheral	115 (43.7)
Axial and peripheral	117 (44.5)
**Extra-articular manifestation**	62 (23.6)

SD = standard deviation; SpA = spondyloarthritis.

**Table 2:** Main clinical and paraclinical findings of patients with spondyloarthritis (N = 263)

Finding	Mean ± SD (range)
**Number of tender joints**	1.3 ± 2.5 (0–16)
**Number of swollen joints**	0.2 ± 0.8 (0–7)
**Frequency of coxitis (%)**	141 (53.6)
**BASMI**	3.9 ± 2.3 (0–10)
**MASES**	1.9 ± 2.8 (0–13)
**HLA B27 allele in %**	56.3
**Disease activity**	
BASG-s	38.1 ± 30.8 (0–100)
BASDAI	49.4 ± 30 (0–100)
ESR in mm/h	36.6 ± 26.3 (2–125)
**Imaging findings**	
mSASSS	15 ± 18.2 (0–72)
**Sacroiliitis in %**	
Grade	1/2 1.9/12.6
Grade	3/4 38.8/46.7
**BASRI**	
Hips	3.3 ± 2.3 (0–4)
Sacro-iliac	3.14 ± 0.9 (0–4)
Spine	3.3 ± 2.3 (0–8)
Total	8.2 ± 3.9 (0–16)
**Functional impact**	
BASFI	45.8 ± 25.7 (0–100)
LI	7.5 ± 6.4 (0–22)

SD = standard deviation; BASMI = Bath ankylosing spondylitis metrology index; MASES = Maastrich ankylosing spondylitis enthesitis score; HLA = human leukocyte antigen; BASG-s = Bath ankylosing spondylitis global score; BASDAI = Bath ankylosing spondylitis disease activity index; ESR = Erythrocyte sedimentation rate; mSASSS = modified stoke ankylosing spondylitis spinal score; BASRI = Bath ankylosing spondylitis radiologic index; BASFI = Bath ankylosing spondylitis functional index; LI = Lequesne index.

**Table 3:** Association of Bath ankylosing spondylitis functional index score and Lequesne index with qualitative parameters in patients with spondyloarthritis

Characteristic	*P* value*	
	BASFI	LI
**Gender**	0.799	0.104
**Level of education**	0.076	0.003
**Professional occupation**	0.010	<0.001
**Classification of SpA**		
Axial	0.157	0.485
Peripheral	0.006	0.021
**HLA B27 allele**	0.153	0.651
**Site of the spinal pain**		
Cervical spine	<0.001	<0.001
Dorsal spine	0.003	0.011
Lumbar spine	0.025	0.371
Buttock pain	0.274	0.3
**Coxo-femoral joint**		
Coxitis	<0.001	<0.001
Bilateral	<0.001	<0.001
Joint limitation	<0.001	<0.001
**BASMI**	<0.001	<0.001
**MASES**	<0.001	0.108
**BASG-s**	<0.001	0.025
**BASDAI**	<0.001	<0.001
**CsDMARDS**		
Yes/no	0.587	0.051
Type	0.280	0.541
**bDMARDS**		
Yes/no	0.812	0.175
Type	0.236	0.282
**Surgical treatment (i.e. THR)**	0.019	<0.001

BASFI = Bath ankylosing spondylitis functional index; LI = Lequesne Index; SpA = spondyloarthritis; HLA = human leukocyte antigen; BASMI = Bath ankylosing spondylitis metrology index; MASES = Maastrich ankylosing spondylitis enthesitis score; BASG-s = Bath ankylosing spondylitis global score; BASDAI = Bath ankylosing spondylitis disease activity index; CsDMARDS = conventional synthetic disease-modifying antirheumatic drugs; bDMARDS = biologic disease-modifying antirheumatic drug; THR = total hip replacement.

* Using one-factor analysis of variance test.

**Table 4:** Correlation of Bath ankylosing spondylitis functional index score and Lequesne index with the quantitative parameters.

Characteristic	Index			
	BASFI		LI	
	r	*P* value*	r	*P* value*
**Disease duration**	0.175	0.007	0.248	<0.001
**BASMI**	0.501	<0.001	0.573	<0.001
**BASG-s**	0.299	<0.001	0.141	0.025
**BASDAI**	0.46	<0.001	0.278	<0.001
**ESR**	0.253	0.001	0.215	0.003
**mSASSS**	0.238	0.001	0.212	0.003
**BASRI**				
Hips	0.322	<0.001	0.610	<0.001
Sacro-iliac	0.277	<0.001	0.307	<0.001
Spine	0.286	<0.001	0.209	0.001
Total	0.385	<0.001	0.468	<0.001
**Grade of sacroiliitis**	0.254	<0.001	0.353	<0.001

BASFI = Bath ankylosing spondylitis functional index; LI = Lequesne index; r = Pearson correlation coefficient; BASMI = Bath ankylosing spondylitis metrology index; BASG-s = Bath ankylosing spondylitis global score; BASDAI = Bath ankylosing spondylitis disease activity index; ESR = erythrocyte sedimentation rate; mSASSS = modified stoke ankylosing spondylitis spinal score; BASRI = Bath ankylosing spondylitis radiologic index.

* Using one-factor analysis of variance test.

**Table 5:** Multiple linear regression of factors associated with Bath ankylosing spondylitis functional index score and Lequesne index

Factor	Index			
	BASFI		LI	
	β (95% CI)	*P* value	β (95% CI)	*P* value
**Profession**	4.20 (−5.66 to 14.08)	0.396	2.72 (0.880 to 4.564)	0.004
**BASMI**	0.70 (−2.60 to 4.01)	0.669	1.15 (0.626 to 1.679)	<0.001
**mSASSS**	0.29 (0.05 to 0.53)	0.016	−0.06 (−0.118 to −0.001)	0.046
**BASDAI**	0.62 (0.42 to 0.83)	<0.001	0.01 (−0.031 to 0.061)	0.524

BASFI = Bath ankylosing spondylitis functional index; LI = Lequesne index; CI = confidence interval; BASMI = Bath ankylosing spondylitis metrology index; mSASSS = modified stoke ankylosing spondylitis spinal score; BASDAI = Bath ankylosing spondylitis disease activity index.
